# Comparing two service delivery models for the prevention of mother-to-child transmission (PMTCT) of HIV during transition from single-dose nevirapine to multi-drug antiretroviral regimens

**DOI:** 10.1186/1471-2458-10-753

**Published:** 2010-12-06

**Authors:** Landry Tsague, Fatima Oliveira Tsiouris, Rosalind J Carter, Veronicah Mugisha, Gilbert Tene, Elevanie Nyankesha, Stephania Koblavi-Deme, Placidie Mugwaneza, Eugenie Kayirangwa, Ruben Sahabo, Elaine J Abrams

**Affiliations:** 1 International Center for AIDS Care and Treatment Programs, Kigali, Rwanda; 2 International Center for AIDS Care and Treatment Programs, Mailman School of Public Health, Columbia University, NY, USA; 3 Treatment and Research AIDS Center (TRAC-Plus), Ministry of Health, Kigali, Rwanda; 4 Centers for Disease Control and Prevention, Kigali, Rwanda; 5 UNICEF-Rwanda

## Abstract

**Background:**

Mother-to-child transmission (MTCT) of HIV has been eliminated from the developed world with the introduction of multi-drug antiretroviral (md-ARV) regimens for the prevention of MTCT (PMTCT); but remains the major cause of HIV infection among sub-Saharan African children. This study compares two service delivery models of PMTCT interventions and documents the lessons learned and the challenges encountered during the transition from single-dose nevirapine (sd-nvp) to md-ARV regimens in a resource-limited setting.

**Methods:**

Program data collected from 32 clinical sites was used to describe trends and compare the performance (uptake of HIV testing, CD4 screening and ARV regimens initiated during pregnancy) of sites providing PMTCT as a stand-alone service (*stand-alone site*) versus sites providing PMTCT as well as antiretroviral therapy (ART) (*full package site*). CD4 cell count screening, enrolment into ART services and the initiation of md-ARV regimens during pregnancy, including dual (zidovudine [AZT] +sd-nvp) prophylaxis and highly active antiretroviral therapy (HAART) were analysed.

**Results:**

From July 2006 to December 2008, 1,622 pregnant women tested HIV positive (HIV+) during antenatal care (ANC). CD4 cell count screening during pregnancy increased from 60% to 70%, and the initiation of md-ARV regimens increased from 35.5% to 97% during this period. In 2008, women attending ANC at *full package *sites were 30% more likely to undergo CD4 cell count assessment during pregnancy than women attending *stand-alone *sites (relative risk (RR) = 1.3; 95% confidence interval (CI): 1.1-1.4). Enrolment of HIV+ pregnant women in ART services was almost twice as likely at *full package *sites than at *stand-alone *sites (RR = 1.9; 95% CI: 1.5-2.3). However, no significant differences were detected between the two models of care in providing md-ARV (RR = 0.9; 95% CI: 0.9-1.0).

**Conclusions:**

All sites successfully transitioned from sd-nvp to md-ARV regimens for PMTCT. *Full package *sites offer the most efficient model for providing immunological assessment and enrolment into care and treatment of HIV+ pregnant women. Strengthening the capacity of *stand-alone *PMTCT sites to achieve the same objectives is paramount.

## Background

Mother-to-child transmission (MTCT) of HIV remains the major route of pediatric HIV infection in sub-Saharan Africa, where over 90% of the 2.1 million children living with HIV reside [1-3]. Since 2000, modest progress has been made towards expanding access to prevention of MTCT (PMTCT) services in sub-Saharan Africa. While access to HIV testing during pregnancy has improved, most PMTCT programs still rely on administration of single-dose nevirapine (sd-nvp) to mothers and babies, a simple, inexpensive, but low efficacy antiretroviral (ARV) regimen to reduce the risk of HIV transmission [3,4]. Since 2004, the World Health Organization (WHO) guidelines have recommended the use of more efficacious multidrug antiretroviral (md-ARV) regimens for PMTCT, including highly active antiretroviral therapy (HAART) for women with advanced disease and short course dual prophylaxis for healthier women not yet eligible for treatment [5]. In November 2009, the WHO issued revised guidelines emphasizing the use of md-ARV for PMTCT as well as the critical need for measuring antenatal CD4 cell counts to determine HAART eligibility [6]. However, only 12% and 28% of HIV+ pregnant women in PMTCT programs received CD4 cell count assessments in 2007 and 2008, respectively, in low- and middle-income countries [1,7]. Moreover, the provision of HAART in antenatal care (ANC) is a challenge due to maternal and child health (MCH) staff shortages, the reliance on medical doctors for HAART initiation and the weak linkages between PMTCT and antiretroviral therapy (ART) services, which often preclude women from being fast-tracked into HIV care and treatment programs [8-11]. Although many sub-Saharan African countries have introduced md-ARV for PMTCT, little has been documented about these experiences [10-13].

Rwanda has a generalized HIV epidemic, 3.6% of women aged 15-49 are living with HIV according to the Demographic and Health Survey conducted in 2005 [14]. It is estimated that approximately 7,700 newborns are at risk of acquiring HIV each year [15]. A national PMTCT program was launched in 1999-2000 at three pilot sites,[16] and coverage expanded to 285 sites nationwide by the end of 2007 [17]. In September 2005, the national PMTCT guidelines were revised to reflect the WHO recommendations that included more effective md-ARV regimens [17]. This study compares two service delivery models for the PMTCT of HIV and compares the uptake of HIV testing, CD4 screening and ARV regimens initiated during pregnancy between 2007 and 2008. It also documents the success of implementation of the program and the challenges encountered during the transition from sd-nvp to md-ARV regimens for PMTCT in 32 sites in Rwanda between July 2006 and December 2008.

## Methods

### Program description

Since 2004, the International Center for AIDS Care and Treatment programs (ICAP) of Columbia University, Mailman School of Public Health (NY, USA), supports the implementation of HIV prevention, care and treatment programs in Rwanda including capacity building of staff, on-site mentoring, renovation of infrastructure, provision of equipment including laboratory machines, technical support to monitoring and evaluation, and operational research. In July 2006, ICAP supported the implementation of PMTCT programs at five health facilities (sites), including four district hospitals (DHs) and one health center (HC), and expanded to 32 sites (four DHs and 28 HCs) by December 2008 in the Western Province (Kibuye and Gisenyi regions) and Kigali, the capital city.

**Figure 1:**
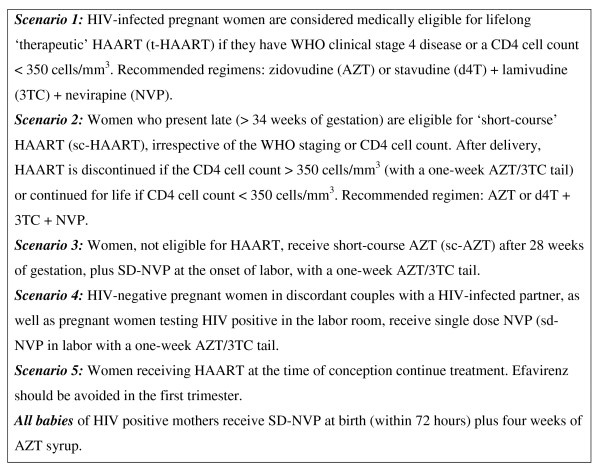
**Guidelines for antiretroviral regimens for the prevention of mother-to-child transmission of HIV in Rwanda, September 2005**.

PMTCT services are routinely implemented within MCH services in Rwanda. Opt-out HIV counseling and testing (CT) is routinely provided in the ANC by trained nurses using sequential rapid HIV tests following the national algorithm, with results available the same-day [18]. Partner testing and couple CT is strongly promoted during pregnancy. The ARV regimens recommended for PMTCT are summarized in Figure 1[19]. WHO clinical staging and CD4 cell count assessment is recommended for all HIV-infected pregnant women. PMTCT staff were trained and mentored to assess the clinical stage and interpret the CD4 cell count test results. At the time of writing this report, the prescription of HAART for eligible patients was still limited to trained physicians, mostly working in ART clinics at district or referral hospitals, and could not be initiated by nurses working in PMTCT programs, predominantly at the HC level.

Two models of PMTCT service delivery were defined: *"full package" *sites where PMTCT and ART services were both located on the same premises and *"stand-alone" *sites where only PMTCT services were available on-site. *Full package *and *stand-alone *sites were authorized to provide md-ARV regimens to pregnant women. However, at the time of writing this report, both *stand-alone *and *full package *sites were providing dual ARV and the sd-NVP regimens, but only *full package *sites offered 'therapeutic' HAART and 'short-course' HAART (Figure 1). Women eligible for HAART in *full package *sites were initiated as soon as possible during pregnancy by the ART physician, whereas *stand-alone *sites referred HAART-eligible women to the nearest ART site for enrolment into ART services and the initiation of treatment.

### Immunologic assessment during pregnancy

In 2006, of the five PMTCT sites, only one had the equipment required to measure CD4 cell counts (FACSCount [Becton Dickinson, San Jose, CA, USA]), the other four sites processed CD4 samples at the nearest laboratory possessing the necessary equipment. CD4 testing requisition was initially only performed for patients within ART clinics since a unique patient identifier (TRACnet number), only provided in the ART clinic, was required by the lab technician before blood could be taken. All pregnant women identified in PMTCT had to be referred to the ART clinic, within the same premises or via a referral (for *stand-alone *sites), to receive a TRACnet ID prior to CD4 testing requisition. In addition, because most CD4 blood samples originated from patients in ART services, pregnant women were therefore asked to come back on a different day, generally within a week after receipt of an HIV positive test result, as this allowed for common batching of blood samples from patients in ART services.

**Figure 2:**
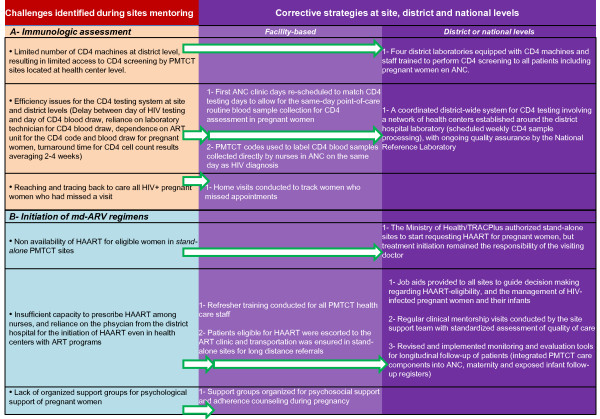
**Challenges and corrective strategies for improving immunologic assessment and initiation of md-ARV regimens during pregnancy**.

Between 2007 and 2008, DH supervisors teamed with mentors from ICAP to support health facilities in assessing and addressing the barriers (Figure 2) to immunologic assessment using an approach described elsewhere [20]. Figure 2 summarizes the changes recommended and gradually implemented at district and site levels.

### Capacity building for the delivering of multidrug ARV (md-ARV) regimens initiation during pregnancy

In 2006, few nurses were trained to administer md-ARV regimens for PMTCT. Refresher training and practical sessions were organized and 297 staff were trained or retrained in 32 sites during the period under review. In each district, the site support staff members worked with district health teams to provide regular on-site mentoring following the completion of formal didactic training.

### Data collection and statistical analysis

We collected the following routine PMTCT program indicators from the program monitoring database for the period July 2006 through December 2008: *number of pregnant women attending first ANC, number of women known to be HIV positive at first ANC, number of women tested for HIV, number of women tested HIV positive, number of male partner counseled and tested for HIV, number of HIV positive women assessed for CD4 cell count during pregnancy, number of women who received CD4 cell count results, number of women with CD4 cell counts < 350 cells/mm3, number of HIV positive women enrolled into HIV care and treatment, number of women with CD4 cell counts < 350 cells/mm3 who initiated HAART for life 'therapeutic' HAART (t-HAART) during pregnancy, number of women who initiated short-course HAART (sc-HAART) for prophylaxis during pregnancy, number of women who initiated dual ARV (AZT/NVP) prophylaxis during pregnancy, number of women who received sd-nvp only (sd-NVP) for PMTCT prophylaxis during pregnancy*. These indicators are routinely collected from site registers, and reported monthly to district health offices for compilation and transmission to the central level. In January 2007, the national program revised the PMTCT program monitoring tools, including registers and monthly summary forms, adding new indicators for CD4 cell count assessments and enrolment in HIV care and treatment programs and HAART initiation. We reported changes over time for each indicator, and using chi-square tests of association with two-sided p-values, we compared the performance of the program across each indicator for the period 2007 - 2008, when comprehensive PMTCT program data was available.

Finally, we compared the two model of service delivery (*full package *vs. *stand-alone*) across key program indicators using relative risks (RR) with associated 95% confidence intervals (CI) for the year 2008 only. The aggregated format of our data did not allow for controlling the effect of clustering across sites. In our analysis, we defined the following ARV regimens options as "more efficacious" ARV regimens (dual ARV, HAART for life, and sc-HAART). Data analysis was conducted using SAS version 9.1 (SAS Institute Inc., Cary, NC, USA). The data used for this analysis were aggregated site level indicators routinely collected by the national PMTCT program, and therefore did not contain any patient identifier. This secondary data analysis received approval from the Columbia University Institutional Review Board (IRB) and was also granted the non-research status (exempted from ethical review) by the Centers for Disease Control and Prevention (CDC), Atlanta, USA.

## Results

### HIV testing uptake among pregnant women and male partners

Between July 2006 and December 2008, 40,674 pregnant women made a first visit to an ANC and were counseled for HIV; 99% accepted HIV testing and 4% tested HIV+; 67% male partners also received HIV testing (Table 1). HIV testing rates, already extremely high at the start of reporting in 2006, increased from 98% to 99% (p < 0.001) from 2007 to 2008. Partner testing rates also increased from 57% in 2007 to 75% in 2008 (p < 0.0001).

**Table 1:** HIV testing, CD4 cell count assessment, and ARV regimens initiated during pregnancy in Rwanda, from July 2006 through to December 2008 (N= 32 sites)

	2006 (Jul-Dec) N (%)	2007 N (%)	2008 N (%)	Total N (%)	p** (2007 vs. 2008)
Total number of sites	14	19	32	-	-
Full package	5 (35.7%)	15 (79%)	18 (56%)	-	-
Counseling and testing of pregnant women in ANC:					
First ANC visit	4,520	15,469	20,685	40,674	-
Known to be HIV positive	NA	135 (0.9%)	291 (1.4%)	426 (1%)	-
Tested for HIV	4,452 (98%)	15,231 (98%)	20,564 (99%)	40,247 (99%)	<0.0001
Tested HIV positive	234 (5%)	741 (5%)	647 (3%)	1,622 (4%)	<0.0001
Partner counseling and HIV testing	NA	8,652 (57%)	15,463 (75%)	24,115 (67%)^$^	<0.0001

CD4 cell count assessment during pregnancy					
Total HIV positive in ANC	234	876	938	2,048	-
Screened for CD4 cell counts^¶^	140 (60%)	610 (70%)	658 (70%)	1,408 (69%)	0.81
Received CD4 cell count results^¶¶^	NA	564 (92%)	647 (98%)	1,211 (96%)^$^	<0.0001
CD4 cell counts < 350 cells/mm^3^	NA	132 (23%)	160 (25%)	292 (24%)^$^	0.59
Enrolment of HIV positive women into care and treatment	NA	180/443 (41%)^@^	564/938 (60%)	744/1381 (54%)^$^	-
HAART initiation for women with CD4 cell counts < 350 cells/mm^3^	NA	114/132 (86%)	127/160 (79%)	241/292 (83%)^$^	0.12

Total HIV positive in ANC	234	876	938	2,048	
All ARV regimens initiated during pregnancy	155 (66%)	667 (76%)	638 (68%)	1460 (71%)	
Dual (AZT/NVP) prophylaxis	55 (35.5%)	386 (58%)	403 (63%)	844 (58%)	
HAART for life	NA	114 (17%)	127 (20%)	241 (16%)	
sd-nvp *only*	100 (64.5%)	34 (5%)	21 (3%)	155 (11%)	
Short-course HAART	NA	133 (20%)	87 (14%)	220 (15%)	
More efficacious ARV regimens (dual ARV, or HAART for life or sc-HAART)	55 (35.5%)	633 (95%)	617 (97%)	1325 (89%)	

^$ ^Percentage calculated based on 2007 and 2008 data only.

NA indicates data not available or not routinely reported in the program monthly report.

^¶ ^Indicates the number of HIV+ pregnant women giving blood for CD4 testing and includes women of known HIV+ status.

^¶¶ ^Indicates the number of HIV+ women receiving CD4 results in a given period and might include women screened in a previous time period. This number is used as a denominator to compute the proportion of women with CD4 cell counts < 350 cells/mm^3^, which reflects the number of women whose results were available and who returned for enrolment in health regimens and subsequent HAART initiation.

^@ ^Data available only for the period from July - December 2007. The denominator was limited to the total number of HIV positive women during the same period.

** Chi-square test, two-sided *p*-value comparing the 2007 to 2008 data only.

### Immunologic assessment and enrolment in ART services during pregnancy

Overall, 69% of 2,048 HIV+ (known and newly identified HIV+) pregnant women were screened for their CD4 cell count during pregnancy, with the proportion increasing from 60% in 2006 to 70% in 2007 and 2008. Additional indicators collected in 2007-2008 showed that 96% of 1,268 women assessed for their CD4 cell count returned to the health facility to retrieve their results and 54% of 1,381 HIV+ women identified from July 2007 through December 2008 were enrolled in ART services. CD4 cell count screening remained at 70% in 2007 and 2008 (p = 0.18), however, the percentage of women receiving their CD4 test results and enrolling in HIV care and treatment programs increased significantly, from 92-98% (p < 0.001) and 41-60% (p = 0.04), respectively (Table 1).

### ARV regimens initiated during pregnancy

Between July 2006 and December 2008, 71% of HIV+ pregnant women received an ARV regimen for PMTCT. The proportion of HIV+ pregnant women initiating more efficacious md-ARV regimens increased from 35.5% in 2006 to 97% in 2008 and women receiving sd-nvp decreased from 64.5% to 3% during the same time-period (Table 1). A total of 292 (24%) women were eligible for HAART in 2007-2008 based on the Rwandan national guidelines (CD4 < 350 cells/mm^3^), and 83% initiated HAART during pregnancy. The proportion of eligible women initiating HAART declined slightly from 86% in 2007 to 79% in 2008 (p = 0.12).

### Comparing the models (*full package *vs. *stand-alone site) *of service delivery

By December 2006, 37.5% of PMTCT sites were providing the *full package *service and 62.5% were *stand-alone *sites; the proportion of *full package *sites increased to 79% by 2007, but decreased to 56% by December 2008 with the opening of new PMTCT sites (Table 1).

From January through December 2008, CD4 assessment of HIV+ pregnant women differed across the models of service delivery with women attending *full package *sites 30% more likely to undergo CD4 cell count assessment than women attending *stand-alone *sites [RR = 1.3; 95% CI: 1.1-1.4] (Table 2). Enrolment of HIV+ pregnant women into ART services was almost twice as likely at *full package *sites than *stand-alone *sites [RR = 1.9; 95% CI: 1.5-2.3]. However, no differences were detected between the two sites, *full package *and *stand-alone*, in initiating treatment for eligible women determined to be eligible for HAART [RR = 0.9; 95% CI: 0.7-1.1]) and providing more efficacious ARV regimens [RR = 0.9; 95% CI: 0.9-1.0].

**Table 2:** CD4 cell count assessment, enrolment into care and treatment and ARV initiated during pregnancy among HIV positive women according to the type of HIV services package, Rwanda, from January through December 2008

	**Full package**^**$**^	Stand-alone	***RR (95% CI)***^***#***^
Sites, N (%)	18 (56%)	14 (44%)	-
Total HIV positive pregnant women (N)	743	195	-
CD4 cell count assessment, N (%):			
Screened for CD4 cell counts^¶^	547 (74%)	111 (57%)	**1.3 (1.1 - 1.4)**
Received CD4 cell count result^¶¶^	536 (98%)	111 (100%)	0.97 (0.96 - 1.0)
CD4 cell count < 350 cells/mm^3^	134 (25%)	26 (23%)	-

Enrolment into care and treatment, N (%)	495 (67%)	69 (35%)	**1.9 (1.5 - 2.3)**
HAART initiation among women with CD4 cell counts < 350 cells/mm^3^, N (%)	105 (78%)	22 (85%)	0.9 (0.7 - 1.1)
Received PMTCT prophylaxis or more efficacious ARV regimens:	511 (96%)	106 (100%)	0.9 (0.9 - 1.0)
Dual (AZT/sd-nvp) prophylaxis	331 (62%)	72 (68%)	-
HAART for life	105 (20%)	22 (21%)	-
Short-course HAART	75 (14%)	12 (11%)	-
sd-nvp *only*	21 (4%)	0 (0%)	-

^$ ^Full package sites provide comprehensive PMTCT and on-site ART services.

^¶ ^The number of HIV+ pregnant women who underwent the CD4 test, including women tested in ANC and those of known HIV+ status who had not initiated HAART before their current pregnancy.

^¶¶ ^The number of HIV+ women receiving CD4 results in a given quarter, including all women who received their results at a follow-up visit during the quarter. This number includes women tested in previous quarters who retrieved their CD4 counts during the indicated quarter.

^# ^Relative Risk (RR) comparing the full package service (PMTCT and ART) as a reference to the stand-alone service; CI: confidence interval.

## Discussion

Until recently, most PMTCT programs still relied on the administration of sd-nvp to mothers and babies but md-ARV regimens have now been implemented. This report documents the successful implementation of md-ARV regimens, including HAART, for HIV+ pregnant women with advanced disease in 32 PMTCT sites in Rwanda. The transition from sd-nvp to more efficacious regimens occurred independently of the model of service delivery (*full package *vs. *stand-alone*) within a 30-month period. To our knowledge, this is one of the first reports to comprehensively document the transition from a traditional sd-nvp regimen to more effective ARV regimens for PMTCT in a resource-limited setting. Despite PMTCT service decentralization to health centers with limited resources and capacities, addressing health systems bottlenecks at site and district levels led to increased numbers of HIV+ women undergoing an immunologic assessment during pregnancy, and improved identification of pregnant women who were eligible for therapy and subsequently initiated on HAART in both *full package *and *stand-alone *sites. However, more effort is needed to ensure effective CD4+ cell count assessments and the enrolment of HIV+ women in ART services in *stand-alone *sites. The finding of such studies will be useful in the design of strategies for implementing the 2009 WHO recommendations into national PMTCT programs.

The number of HIV+ pregnant women who were screened for CD4 cell counts during pregnancy (up to 70% in 2008) and who received their results (up to 98% in 2008) significantly increased following the introduction of changes to the CD4 testing system at both the site and district levels. One major innovation was the same-day point-of-care blood sample taken from HIV+ pregnant women in ANC clinics. The importance of same-day CD4 cell count testing was also highlighted in two studies from South Africa reporting an uptake rate of over 97% for CD4 cell count screening during pregnancy [10,11]. Although most sites in this program sent samples to off-site laboratories mainly at the DH level for CD4 measurement, it could be anticipated that with the implementation of a weekly sample processing system, the turnaround time for CD4 cell count results would be reduced. Our data further suggested that despite the improvements in CD4 cell count assessments over time, women in *full package *sites were more likely to undergo immunologic screening than those in *stand-alone *sites. Although our study did not investigate the reasons for such a difference, it could be hypothesized that in 2008 the addition of 13 new *stand-alone *sites, which likely possessed ineffective CD4 testing systems, reduced the overall performance of *stand-alone *sites.

We found that approximately one quarter of pregnant women were eligible for HAART, based on CD4 cell counts ≤ 350 cells/mm^3^. This is two-fold lower than the 55% reported in a study in the Western Cape, South Africa,[11] suggesting that most pregnant women are diagnosed earlier in the course of HIV infection in our clinical settings. In addition, in our findings, a high proportion (85%) of eligible pregnant women initiated HAART with no significant differences between the models of service delivery. Although women in *full package *sites were more likely to undergo CD4 testing, women who received testing and were determined to be eligible were just as likely to initiate HAART in *stand-alone *sites as in *full package *sites in 2008, suggesting that the changes that were introduced had a positive impact, as summarized in Figure 2.

In our report, the uptake of HAART among women identified as eligible was found to be higher than the 51% reported in five sites in the Western Cape [11] and the 75% reported in Gauteng province, [10] South Africa. Similar to the findings in the Western Cape, our study revealed that the model of service delivery did not influence the rate of HAART initiation during pregnancy [11]. The high uptake of HAART among eligible women identified in our study might be attributable to a number of innovations including improved psychosocial support, escorting eligible women to the ART clinic and liaising with social workers to track women who missed appointments. A tracking system for pregnant women was deemed more feasible in our setting given the small number of HAART-eligible women compared with a higher prevalence setting like South Africa. Furthermore, denial and fear of disclosure to sexual partners was reported as a limiting factor for effective treatment in South Africa, whereas the high rate of partner testing and the roll-out of psychosocial support structures (e.g., associations of people living with HIV and AIDS, and support groups for HIV+ patients) over the past two years could have mitigated the impact of this factor in our setting. Finally, according to the 2008 Rwanda Interim Demographic and Health (I-DHS) survey, about 96% of pregnant women attend at least one ANC visit during their pregnancy; most of them attending ANC for the first time before the end of the second trimester, giving enough time for those women tested HIV infected to start the ARV prophylaxis or treatment.

Health care providers in *full package *sites may be prompted to actively enroll women found to be eligible for HAART in the ART program, given that HAART initiation could be done in the same facility. We could hypothesize therefore, that making HAART available in *stand-alone *sites and accessible for eligible pregnant women may provide a motivation for providers to undertake CD4 cell count screening and enroll HIV+ and eligible pregnant women into ART services. By doing so, it would further reduce the burden on pregnant women who were asked to travel, in some cases, long distances to reach the nearest ART site.

We noted, however, that the two models of service delivery did not differ with regard to HAART initiation among eligible pregnant women. This results from the effective system for referral of eligible pregnant women from *stand-alone *sites to the nearest *full package *site, coupled with the availability of doctors from the DH who usually visit *full package *sites on a weekly basis for HAART initiation among new HIV+ patients. As PMTCT services continue to be scaled-up at the district level, the *"flying doctors" *approach might not be cost-effective and sustainable in resource limited settings. Task-shifting HAART initiation to nurses is therefore necessary and this has been successful performed in various settings [21-24]. Rwanda adopted in 2009 a national policy on task-shifting for HIV services and its implementation will contribute to improve and sustain the timely initiation of HAART, particularly among eligible HIV+ pregnant women.

Some key factors for an effective transition from sd-nvp to md-ARV regimens include among others the capacity building and regular mentoring of nurses, the routine assessment of the quality of care provided by nurses during the transition to md-ARV regimens, the full integration of PMTCT care components into MCH services, the strengthening of the CD4 testing capacity at district level, the reorganizing of services at site level with an emphasis on effective linkages between PMTCT and ART services, and the high level commitment and leadership of the Rwandan government's to improving maternal and child survival, including virtual elimination of pediatric HIV infection.

The analysis carried out in this study was subject to some limitations. A recent analysis of routine PMTCT data in South Africa concluded that there were major weaknesses in the completeness and accuracy of this data which precluded its use in the tracking of process performance of facilities,[25] however, we believe that the routine PMTCT data in Rwanda is of good quality due to the implementation of a rigorous quality assurance process whereby aggregate reports are verified against program registers each month by site support staff. In addition, by focusing the analysis on antenatal services, findings are limited to the initiation of PMTCT regimens during pregnancy, and do not address maternal adherence to ARV regimens during pregnancy, maternal and infant ARV uptake at delivery, or the overall impact of PMTCT interventions on maternal and child health. Also, the improvements introduced in the routine data collection system in 2007 have likely increased reporting on the uptake of various PMTCT interventions, compared with 2006. We were unable to account for clustering of patients within clinics in our analysis, thus underestimating the variance associated with each program indicator. Finally, since several interventions occurred simultaneously, it is difficult to determine the relative importance of each intervention on program performance.

## Conclusions

This report has demonstrated that regardless of the model of PMTCT service delivery, it is feasible to transition from sd-nvp regimen to more effective md-ARV regimen in a resource limited, public sector PMTCT program. However, more efforts are needed to ensure effective CD4+ cell count assessment and the enrolment of HIV+ women in ART services in *stand-alone sites*, and task-shifting is necessary to ensure the timely initiation and follow-up of HAART among pregnant women. These findings provide valuable insight which can be applied to the design of effective service delivery models for the transition from sd-nvp to md-ARV within national PMTCT programs in resource limited settings, accelerating progress towards virtual elimination of MTCT of HIV by 2015 [26].

## Competing interests

The authors declare that they have no competing interests.

## Authors' contributions

LT, FT, RC, VM, GT, EM, SK, PM, EK, RS, EJA contributed to the design of the program and the development of the concept for this manuscript. LT, FT, RC wrote the first draft of the manuscript. SK reviewed the laboratory section of the methodology. LT, RC, FT, EJA conducted data analysis and interpretation. And all the authors reviewed the final manuscript.

## Authors' information

At the time of this report, Landry Tsague was the Clinical Director at the International Center for AIDS Care and Treatment Program (ICAP)/Columbia University, Kigali, Rwanda. He is now with UNICEF in Rwanda.

## Pre-publication history

The pre-publication history for this paper can be accessed here:

http://www.biomedcentral.com/1471-2458/10/753/prepub

## References

[B1] World Health Organization, Joint United Nations Programs on HIV/AIDS, United Nations Children's Fund Towards universal access: scaling up priority HIV/AIDS interventions in the health sector: 2008 progress report 2008 World Health Organization

[B2] Kourtis AP Lee FK Abrams EJ Jamieson DJ Bulterys M Mother-to-child transmission of HIV-1: timing and implications for prevention Lancet Infect Dis 2006 6 726 732 10.1016/S1473-3099(06)70629-6 17067921

[B3] De Cock KM Fowler MG Mercier E de Vincenzi I Saba J Hoff E Alnwick DJ Rogers M Shaffer N Prevention of mother-to-child HIV transmission in resource-poor countries: translating research into policy and practice JAMA 2000 283 1175 1182 10.1001/jama.283.9.1175 10703780

[B4] Guay LA Musoke P Fleming T Bagenda D Allen M Nakabiito C Sherman J Bakaki P Ducar C Deseyve M Intrapartum and neonatal single-dose nevirapine compared with zidovudine for prevention of mother-to-child transmission of HIV-1 in Kampala, Uganda: HIVNET 012 randomised trial. [see comment] Lancet 1999 354 795 802 10485720 10.1016/S0140-6736(99)80008-7

[B5] Antiretroviral Drugs for Treating Pregnant Women and Preventig HIV Infection in Infants: Towards Universal Access. Recommendations for a Public Health Approach http://www.who.int/hiv/pub/guidelines/pmtctguidelines2.pdf --- Either ISSN or Journal title must be supplied.

[B6] World Health Organization Rapid advice: use of antiretroviral drugs for treating pregnant women and preventing HIV Infection in infants 2009 --- Either ISSN or Journal title must be supplied. PMC3535284 23293399

[B7] World Health Organization, Joint United Nations Programs on HIV/AIDS, United Nations Children's Fund Towards universal access: scaling up priority HIV/AIDS interventions in the health sector: 2009 progress report 2009 World Health Organization

[B8] Myer L Rabkin M Abrams EJ Rosenfield A El-Sadr WM Focus on women: linking HIV care and treatment with reproductive health services in the MTCT-Plus Initiative Reprod Health Matters 2005 13 136 146 10.1016/S0968-8080(05)25185-6 16035607

[B9] UNICEF/WHO Antenatal care in developing countries: promises, achievements and missed opportunities http://www.who.int/reproductive-health/publications/antenatal_care/ --- Either ISSN or Journal title must be supplied.

[B10] van der Merwe K Chersich MF Technau K Umurungi Y Conradie F Coovadia A Integration of antiretroviral treatment within antenatal care in Gauteng Province, South Africa J Acquir Immune Defic Syndr 2006 43 577 581 17031321 10.1097/01.qai.0000243099.72770.d2

[B11] Stinson K Myer L Boulle A An evaluation of approaches to the initiation of antiretroviral therapy during pregnancy among HIV-infected women in Cape-Town 2008 Cape Town: University of Cape Town

[B12] Killam WP Tambatamba BC Chintu N Rouse D Stringer E Bweupe M Yu Y Stringer JS Antiretroviral therapy in antenatal care to increase treatment initiation in HIV-infected pregnant women: a stepped-wedge evaluation Aids 2009 24 85 91 10.1097/QAD.0b013e32833298be 19809271

[B13] Tchendjou P Same-Ekobo C Nga A Tejiokem M Kfutwah A Nlend AN Tsague L Bissek AC Ekoa D Orne-Gliemann J Effectiveness of multidrug antiretroviral regimens to prevent mother-to-child transmission of HIV-1 in routine public health services in Cameroon PLoS One 5 e10411 10.1371/journal.pone.0010411 20454459 PMC2861601

[B14] Institut National de la Statistique du Rwanda (INSR) and ORC Macro Rwanda Demographic and Health Survey 2005 2006 Calverton, Maryland, U.S.A.: INSR and ORC Macro

[B15] Minisitry of Health Treatment and Reseach on AIDS Center (TRAC) Scale up Plan of the Prevention of Mother to Child Transmission of HIV (PMTCT) Program in Rwanda (2007-2011) 2007 --- Either ISSN or Journal title must be supplied.

[B16] Rutenberg N Baek C Kaliba S Evaluation of United Nations-Supported Pilot Projects for the Prevention of Mother-to-Child Transmission of HIV 2003 (UNICEF ed. New-York: UNICEF)

[B17] Treatment and Research AIDS Center (TRAC) Rapport annuel du TRAC 2007 Health Mo ed. Kigali, Rwanda

[B18] Minisitry of Health, Treatment and Research AIDS Center (TRAC) Normes et directives nationales du Conseils et dépistage volontaire et Prévention de la Transmission du VIH de la Mère à l'Enfant 2006 Kigali, Rwanda

[B19] Ministry of Health, Treatment and Research AIDS Center (TRAC) Guide de prise en charge des personnes infectees par le VIH au Rwanda 2007 Kigali, Rwanda

[B20] The International Center for AIDS Care and Treatment Programs (ICAP) at Columbia University's Mailman School of Public Health in New York City Clinical Systems Mentorship: The ICAP Guide to Site Support 2007 --- Either ISSN or Journal title must be supplied.

[B21] Shumbusho F Price J Binagwaho A Turate I Task shifting to achieve universal access to HIV care: Evaluation of a pilot program of antiretroviral treatment initiation in Rwanda PEPFAR Implementers Meeting, Uganda 2008

[B22] Mills K Clutterbuck DJ Seitio O Sebego M Riley A Antiretroviral treatment roll-out in a resourceconstrained setting: Capitalizing on nursing resources in Botswana WHO Bulletin 2007 85 10.2471/BLT.06.033076 PMC2636363 17768505

[B23] Gimbel-Sherr SO Micek MA Gimbel-Sherr KH Koepsell T Hughes JP Thomas KK Pfeiffer J Gloyd SS Using nurses to identify HAART eligible patients in the Republic of Mozambique: results of a time series analysis Hum Resour Health 2007 5 7 10.1186/1478-4491-5-7 17328804 PMC1817650

[B24] Bolton-Moore C Mubiana-Mbewe M Cantrell RA Chintu N Stringer EM Chi BH Sinkala M Kankasa C Wilson CM Wilfert CM Clinical outcomes and CD4 cell response in children receiving antiretroviral therapy at primary health care facilities in Zambia Jama 2007 298 1888 1899 10.1001/jama.298.16.1888 17954540

[B25] Mate KS Bennett B Mphatswe W Barker P Rollins N Challenges for Routine Health System Data Management in a Large Public Programme to Prevent Mother-to-Child HIV Transmission in South Africa PLoS ONE 2009 4 5 e5483 10.1371/journal.pone.0005483 19434234 PMC2677154

[B26] UINCEF UNAIDS WHO UNEFPA UNESCO Children and AIDS: Fifth Stocktaking Report 2010 http://www.unicef.org/aids/files/Children_and_AIDS-Fifth_Stocktaking_Report_2010_LoRes_EN(2).pdf

